# The Role of Self‐Compassion on Self‐Care With the Mediating Role of Illness Perception in Patients With Multiple Sclerosis Referred to Sina Hospital: Based on the Leventhal Model

**DOI:** 10.1002/brb3.71772

**Published:** 2026-09-17

**Authors:** Ali Bagheri, Amir Sam Kianimoghadam, Zahra Ebadi, Abbas Masjedi‐Arani, Maryam Amini‐Fasakhoudi

**Affiliations:** ^1^ Department of Clinical Psychology, School of Medicine Shahid Beheshti University of Medical Sciences Tehran Iran; ^2^ Multiple Sclerosis Research Center, Neuroscience Institute Tehran University of Medical Sciences Tehran Iran

**Keywords:** multiple sclerosis, self‐compassion, self‐care, illness perception, quality of life, structural equation model

## Abstract

**Background:**

Multiple sclerosis (MS) is a chronic neurodegenerative disorder associated with diverse physical and psychological challenges. Effective self‐care behaviors are essential for managing symptoms and improving quality of life. Psychological constructs such as self‐compassion and illness perception are key determinants of self‐management in chronic illness.

**Objective:**

This study investigated the role of self‐compassion in promoting self‐care behaviors among patients with MS, emphasizing the mediating role of illness perception based on Leventhal's Common‐Sense Model.

**Methods:**

A cross‐sectional analytical study was conducted among 360 patients with MS referred to Sina Hospital, Tehran. Participants completed the *Self‐Care of Chronic Illness Inventory (Version 4.c)*, *Revised Illness Perception Questionnaire (IPQ‐R)*, and *Self‐Compassion Scale (SCS)*. Structural equation modeling (SEM) was used to examine direct and indirect relationships between self‐compassion, illness perception dimensions, and self‐care behaviors. Model fit was evaluated using *χ* ^2^/df = 2.52, CFI = 0.843, TLI = 0.825, RMSEA = 0.065, and SRMR = 0.084.

**Results:**

Self‐compassion had a significant positive direct effect on self‐care (*β* = 0.30, *p* = 0.006). Indirect effects were also found through personal control (*β* = 0.15, *p* = 0.004), emotional representation (*β* = 0.22, *p* = 0.011), and illness coherence (*β* = 0.15, *p* = 0.030). Other illness perception dimensions, including treatment control and timeline beliefs, did not show significant mediation effects (*p* > 0.05).

**Conclusion:**

Self‐compassion promotes self‐care behaviors in MS patients both directly and indirectly by enhancing adaptive illness perceptions—particularly personal control, illness coherence, and emotional balance. Interventions aimed at fostering self‐compassion and reframing maladaptive illness perceptions may strengthen disease self‐management and psychological well‐being.

## Graphical abstract

This graphical abstract illustrates the mediating role of illness perception in the relationship between self‐compassion and self‐care among patients with multiple sclerosis (MS). Higher self‐compassion enhances self‐care both directly and indirectly through adaptive illness perceptions—specifically greater personal control, stronger illness coherence, and more balanced emotional representation—highlighting key psychological pathways for improving MS self‐management.

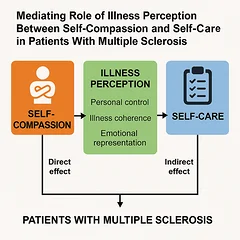

## 1 Introduction

Multiple sclerosis (MS) is an immune‐mediated, chronic neurodegenerative disorder of the central nervous system (CNS) characterized by inflammatory lesions, demyelination, and progressive axonal damage (Walton et al. 2020). Its clinical presentation is heterogeneous, including symptoms such as fatigue, visual disturbances, muscle weakness, bladder and bowel dysfunction, balance impairments, cognitive decline, and psychological manifestations such as depression and anxiety (Ziaie et al. 2024). Globally, MS primarily affects young adults between the ages of 20 and 40, with a higher incidence in women than in men (Vaughn et al. 2019).

MS can be categorized into four main subtypes—clinically isolated syndrome (CIS), relapsing‐remitting MS (RRMS), secondary progressive MS (SPMS), and primary progressive MS (PPMS)—each reflecting a distinct disease trajectory. CIS represents the initial neurological event involving inflammation or demyelination. RRMS, which accounts for 85%–90% of cases, is characterized by relapses followed by partial or complete recovery, and it often progresses to SPMS, marked by continuous neurological decline without remission. Approximately 10%–15% of patients develop PPMS, which involves gradual deterioration without distinct relapses or recoveries (Shriwash et al. 2024).

Epidemiological evidence indicates a rising prevalence of MS worldwide, with approximately one new diagnosis occurring every 5 min, contributing to an estimated 2.8 million affected individuals globally (Khutsishvili et al. 2024). Although Iran was historically considered a low‐risk region based on its geographic coordinates (32° N, 53° E), recent data show a substantial increase in prevalence. In 2020, the pooled prevalence of MS in Iran was approximately 100 cases per 100,000 population—representing a threefold increase compared with estimates from 2019—highlighting a marked upward trend in incidence (Mirmosayyeb et al. 2022).

Individuals affected by MS must continuously adapt to the chronic and unpredictable challenges imposed by the disease (Faraji et al. 2018). Although MS is not curable, disease‐modifying therapies (DMTs) have proven effective in reducing relapse rates and slowing disease progression. However, adherence to these treatments remains a major challenge, as many patients discontinue DMTs prematurely due to fluctuating symptoms, extended remission periods, treatment side effects, or a perceived lack of improvement (Wilski et al. 2019). Beyond pharmacological management, consistent engagement in self‐care practices represents a crucial strategy for coping with the physical and psychological burdens of MS. Sustained self‐care not only enhances quality of life but also helps prevent or delay both acute and long‐term complications associated with the disease. Evidence further suggests that inadequate self‐care in MS is linked to higher relapse rates, faster disability progression, and reduced quality of life (Oliveira‐Kumakura et al. 2019; Khodaveisi et al. 2018).

The Middle‐Range Theory of Self‐Care of Chronic Illness (MRT‐SCCI) conceptualizes self‐care as a dynamic and multidimensional process involving three interrelated components: maintenance, monitoring, and management. These components reflect the ways individuals maintain health, detect bodily changes, and respond to emerging symptoms (Riegel et al. 2018). The theory emphasizes that effective self‐care depends not only on physiological awareness but also on psychological and contextual factors such as self‐efficacy, prior experiences, social support, and access to healthcare (Riegel et al. 2019). This framework was adopted in the present study because it provides a clear structure for understanding how individuals with MS engage in self‐regulatory behaviors to manage a complex chronic condition.

Complementing this perspective, Leventhal's Common‐Sense Model (CSM) of illness self‐regulation explains how patients’ cognitive and emotional representations of their illness shape coping strategies and health behaviors. According to this model, individuals interpret their illness through beliefs about its identity, duration, causes, consequences, controllability, and coherence, while also experiencing affective responses such as fear, anxiety, or distress. Together, these representations influence how patients evaluate and adjust their coping efforts over time (Hagger and Orbell 2022). Empirical evidence shows that adaptive illness perceptions—marked by a sense of control, understanding, and emotional balance—are associated with better adherence and self‐care, whereas maladaptive perceptions predict poorer psychological and behavioral outcomes (Leventhal et al. 2016).

Parallel to illness perception, self‐compassion has been increasingly recognized as a psychological resource influencing health behaviors and coping in chronic illness contexts. Defined as a kind, nonjudgmental, and mindful attitude toward oneself in times of suffering or failure, self‐compassion comprises three interrelated elements: self‐kindness versus self‐judgment, common humanity versus isolation, and mindfulness versus over‐identification (Neff 2023). Individuals with higher levels of self‐compassion demonstrate greater emotional regulation, lower psychological distress, and enhanced resilience, enabling more adaptive responses to the multifaceted challenges of MS (Sirois et al. 2019).

Although recent studies have independently highlighted the importance of self‐compassion and illness perception in promoting psychological adjustment and self‐care among patients with chronic illnesses, their combined and mediating mechanisms remain unexplored in MS. Evidence indicates that self‐compassion enhances adaptive coping and treatment adherence (Baxter and Sirois 2025; Eghbali et al. 2022), while illness perception significantly influences self‐management and quality of life (Timkova et al. 2021; Huang et al. 2024). However, to date, no study has simultaneously examined the interrelationship among self‐compassion, illness perception, and self‐care, particularly within non‐Western cultural contexts where sociocultural factors may uniquely shape these associations. Addressing this research gap, the present study investigates the effect of self‐compassion on self‐care among Iranian patients with MS, emphasizing the mediating role of illness perception within Leventhal's self‐regulation framework.

## 2 Methods

### 2.1 Study Design and Setting

A cross‐sectional analytical study was conducted at the Neurology Department of Sina Hospital, Tehran, Iran, between August 2024 and January 2025. The hypothesized relationships were examined using structural equation modeling (SEM).

### 2.2 Participants and Procedures

The study population comprised patients with MS attending routine outpatient visits at Sina Hospital. Participants were recruited through in‐person invitations using a convenience sampling approach during the study period. Eligible patients received a brief explanation of the study aims and procedures and, after providing written informed consent, completed the questionnaires anonymously in a quiet and private setting.

To ensure anonymity, no identifying information (e.g., name, national ID, or medical record number) was collected. Questionnaires were completed individually, and participants were explicitly informed that their responses would remain confidential and would not affect their medical care. A researcher remained available only to address procedural questions (e.g., instructions for completion) and did not provide guidance on item content. Upon completion, participants placed the questionnaires in sealed envelopes, which were then collected. To reduce order effects, questionnaires were administered in a randomized order. The average completion time was approximately 30 min.

Inclusion criteria were (1) MS diagnosis confirmed by a neurologist according to the McDonald criteria, (2) at least 6 months since diagnosis, (3) ability to read and write in Persian, (4) adequate cognitive and physical capacity to complete questionnaires independently (EDSS ≤ 6), (5) age 18–60 years, and (6) voluntary participation. Exclusion criteria included withdrawal at any stage, severe distress during data collection, or questionnaires with incomplete or inconsistent responses. A 10% attrition rate was anticipated based on prior studies. Of 400 eligible patients approached, 360 completed the questionnaires (response rate = 90%).

Adequate sample size for SEM was determined using established recommendations for models of comparable complexity and Soper's (2020) a priori calculator. Guidelines typically recommend 200–400 participants for complex SEM, depending on model complexity and the number of estimated parameters (Kline 2016; Hair et al. 2019). Based on 83 observed variables, a medium effect size (0.30), power (0.80), and alpha (0.05), the minimum required sample size was estimated at approximately 340 (Soper 2020). Accordingly, 400 patients were recruited, and after applying predefined exclusion criteria, the final sample (*n* = 360) exceeded the minimum requirement and was considered sufficient to test the proposed mediation model.

### 2.3 Instruments

#### 2.3.1 Sociodemographic Questionnaire

A researcher‐developed questionnaire was used to collect participants’ demographic information, including age, gender, educational level, and marital status.

#### 2.3.2 Persian Version of Self‐Care of Chronic Illness Inventory Version 4.c (Persian SC‐CII v4.c)

The Self‐Care of Chronic Illness Inventory Version 4.c (SC‐CII v4.c) is a self‐report measure grounded in the MRT‐SCCI and includes three subscales: self‐care maintenance (seven items), self‐care monitoring (five items), and self‐care management (seven items). Items are rated primarily on a 1–5 ordinal scale, with selected management items allowing a 0 response option (Riegel et al. 2018). Subscale scores are standardized to 0–100, with higher scores indicating better self‐care and a cutoff of 70 representing adequate self‐care (Riegel et al. 2018). The instrument has demonstrated acceptable psychometric properties in both original and Iranian validations (Riegel et al. 2018, Bagheri et al. unpublished manuscript). In the current study, Cronbach's alpha was 0.80 (maintenance), 0.79 (monitoring), and 0.82 (management).

#### 2.3.3 Revised Illness Perception Questionnaire (IPQ‐R)

Illness perceptions were assessed using seven subscales from the Persian adaptation of the IPQ‐R, excluding Identity and Causal Attribution: timeline acute/chronic, timeline cyclical, consequences, personal control, treatment control, illness coherence, and emotional representation. Items are rated on a 5‐point Likert scale (1 = *strongly disagree* to 5 = *strongly agree*), with some items reverse scored (Moss‐Morris et al. 2002). The IPQ‐R has shown strong psychometric performance, with construct validity supported by confirmatory factor analysis (CFA) confirming the seven‐factor structure (Moss‐Morris et al. 2002). The Persian version has shown strong validity and reliability in Iranian samples (Bagheri et al. 2025). In this study, internal consistency across subscales ranged from *α* = 0.78 to 0.83.

#### 2.3.4 Self‐Compassion Scale (SCS)

Self‐compassion was measured using the 26‐item SCS, covering six subscales that reflect three components: self‐kindness versus self‐judgment, common humanity versus isolation, and mindfulness versus over‐identification. Items are rated on a 5‐point Likert scale (1 = *almost never* to 5 = *almost always*), and negatively keyed items are reverse scored before computing subscale means and the total mean score (Neff 2003). The original SCS shows strong reliability (*α* = 0.90; test–retest *r* = 0.93) (Neff 2003), and the Persian version has also demonstrated good psychometric properties (overall *α* = 0.78; subscales *α* = 0.79–0.93) (Azizi et al. 2013). In the current sample, Cronbach's alpha for the total scale was 0.79.

### 2.4 Statistical Analysis

Data screening was conducted to evaluate missingness and data quality prior to modeling. Missing responses were examined for amount and pattern; given that missingness was minimal and did not suggest a systematic pattern, complete‐case analysis (listwise deletion) was used, and only participants with fully completed questionnaires were included in SEM.

Descriptive statistics were computed to summarize participants’ characteristics and describe the distributions of study variables. SEM was then conducted to test the hypothesized pathways between self‐compassion (independent variable), illness perception dimensions (mediators), and self‐care behaviors (dependent variable) in patients with MS.

Before estimating the measurement and structural models, assumptions relevant to SEM (including distributional characteristics, residual patterns, and multicollinearity) were examined using standard diagnostics. Multicollinearity was evaluated using tolerance and VIF indices (tolerance > 0.10; VIF < 10) (Tabachnick and Fidell 2013; Field 2018).

A CFA was performed to evaluate the measurement model. Model fit was assessed using *χ* ^2^/df, CFI, TLI, RMSEA, and SRMR, applying conventional cutoffs (CFI/TLI ≥ 0.90; RMSEA/SRMR ≤ 0.08) (Kline 2016). Standardized factor loadings were inspected, and Composite reliability (CR) and average variance extracted (AVE) were calculated to evaluate internal consistency and convergent validity (CR ≥ 0.70; AVE ≥ 0.50) (Hair et al. 2021). Discriminant validity was assessed using the Fornell–Larcker criterion (Fornell and Larcker 1981).

The structural model was then estimated to test the direct effect of self‐compassion on self‐care and indirect effects through illness perception dimensions, consistent with Leventhal's CSM of self‐regulation. Indirect effects were tested via bootstrapping (5000 resamples) to generate bias‐corrected 95% confidence intervals. Non‐significant paths were trimmed to improve model parsimony. Analyses were conducted using IBM SPSS Statistics 26 and IBM SPSS AMOS 24, with statistical significance set at *p* < 0.05.

### 2.5 Ethical Considerations

The study protocol was approved by the Research Ethics Committee of the School of Medicine, Shahid Beheshti University of Medical Sciences (IR.SBMU.MSP.REC.1402.570). All participants provided written informed consent and were informed that participation was voluntary and that they could withdraw at any time without consequences for their care.

## 3 Results

### 3.1 Characteristics of the Participants

Most of the 360 participants were middle‐aged (*M* = 39.38, SD = 9.93), female (55.3%), and 47.2% were married. Regarding education, 21.4% had a high school diploma or lower, 19.4% held an associate degree, 21.1% had a bachelor's degree, 18.6% had a master's degree, and 19.4% held a doctoral degree. Sociodemographic characteristics are summarized in Table 1.

**TABLE 1:** Sociodemographic characteristics of the participants.

Variable	*n* (%) or M (SD)
Age, years	39.38 (9.93)
Education	
High school diploma or lower	77 (21.4)
Associate's degree	70 (19.4)
Bachelor's degree	76 (21.1)
Master's degree	67 (18.6)
Doctoral degree	70 (19.4)
Gender	
Female	199 (55.3)
Male	161 (44.7)
Marital status	
Married	170 (47.2)
Single	190 (52.8)

*Note*: *N* = 360. Percentages may not total 100 due to rounding. Age is presented as M (SD).

The mean scores for the study variables were as follows: self‐compassion (*M* = 3.01, SD = 0.36), timeline acute (*M* = 3.10, SD = 0.54), consequences (*M* = 2.82, SD = 0.50), personal control (*M* = 3.14, SD = 0.54), treatment control (*M* = 3.15, SD = 0.54), illness coherence (*M* = 3.16, SD = 0.49), timeline cyclical (*M* = 2.91, SD = 0.55), emotional representation (*M* = 3.12, SD = 0.49), and self‐care (*M* = 3.26, SD = 0.42).

### 3.2 Assessment and Validation of Key Assumptions for SEM

Bivariate scatterplots indicated approximately linear relationships among self‐compassion, the mediators, and self‐care, with no observable nonlinear or curvilinear patterns (see Supporting Information File 1). Pearson correlation coefficients further confirmed significant positive linear associations among these variables (see Table 2).

**TABLE 2:** Pearson correlations among self‐compassion, mediators, and self‐care.

	1	2	3	4	5	6	7	8	9
1. Self‐compassion	—	−0/016	0/052	0.185^**^	0/097	0.219^**^	0/018	0.366^**^	0.352^**^
2. Timeline acute	−0/016	—	0.304^**^	0/074	0.351^**^	0/095	0.338^**^	0.111^*^	−0.130^*^
3.Consequences	0/052	0.304^**^	—	0.152^**^	0.223^**^	0.145^**^	0.232^**^	0/078	0/015
4. Personal control	0.185^**^	0/074	0.152^**^	—	0.147^**^	0.376^**^	0.172^**^	0.309^**^	0.379^**^
5. Treatment control	0/097	0.351^**^	0.223^**^	0.147^**^	—	0.204^**^	0.290^**^	0.171^**^	0/067
6. Illness coherence	0.219^**^	0/095	0.145^**^	0.376^**^	0.204^**^	—	0.153^**^	0.413^**^	0.412^**^
7. Timeline cyclical	0/018	0.338^**^	0.232^**^	0.172^**^	0.290^**^	0.153^**^	—	0.111^*^	−0/039
8. Emotional representation	0.366^**^	0.111^*^	0/078	0.309^**^	0.171^**^	0.413^**^	0.111^*^	—	0.461^**^
9. Self‐care	0.352^**^	−0.130^*^	0/015	0.379^**^	0/067	0.412^**^	−0/039	0.461^**^	—

*Note*: *N* = 360. Values are Pearson's *r*. Two‐tailed tests.

^*^p < 0.05, ^**^ *p* < 0.01.

Multivariate normality was assessed using Mardia's coefficient and Mahalanobis distance. Mardia's multivariate kurtosis was 31.17 (c.r. = 4.36), indicating a mild departure from multivariate normality; however, SEM estimates were obtained using bootstrapping, which is relatively robust to non‐normality.

Mahalanobis distances were compared against the *χ* ^2^ critical value for df = 8 at *p* = 0.001 (*χ* ^2^ = 26.12). The maximum distance (45.22) exceeded this threshold for only four cases (IDs 177, 233, 272, and 296; < 1.1% of the sample), suggesting a very small proportion of multivariate outliers unlikely to influence results under bootstrapped estimation.

At the univariate level, skewness and kurtosis for all observed variables were within ± 2, and the Kolmogorov–Smirnov tests were non‐significant (*p* > 0.05), consistent with approximately normal distributions; histogram and *Q–Q* plot inspections were congruent with these findings (Supporting Information File 2).

Homoscedasticity was evaluated via the standardized residuals‐versus‐predicted plot, which showed a random pattern around zero with most residuals within ± 3 SD (Supporting Information File 3).

Multicollinearity diagnostics indicated no concerns (tolerance = 0.723–0.864; VIF = 1.157–1.383; condition indices < 30) (Supporting Information File 4). Collectively, these diagnostics supported the adequacy of the data for SEM to test the proposed pathways among self‐compassion, illness perceptions, and self‐care in patients with MS.

### 3.3 Measurement Model Evaluation

Prior to testing the hypothesized structural relationships, a CFA was conducted to assess the reliability and validity of the measurement model. All standardized factor loadings were statistically significant (*p* < 0.001) and ranged from 0.42 to 0.83, exceeding the minimum acceptable threshold of 0.40 (see Table 3).

**TABLE 3:** Composite reliability (CR), average variance extracted (AVE), and square root of AVE (√AVE) by scale.

Scale	CR	AVE	√AVE
Timeline cyclical	0.797	0.497	0.705
Timeline acute	0.825	0.443	0.665
Consequences	0.803	0.409	0.640
Personal control	0.812	0.419	0.648
Treatment control	0.801	0.449	0.670
Illness coherence	0.786	0.428	0.654
Emotional representation	0.838	0.469	0.685
Self care	0.645	0.379	0.616
Self compassion	0.636	0.227	0.477

*Note*: Values are computed from standardized.

Composite reliability (CR) values ranged from 0.64 (F1: self‐compassion composite) to 0.84 (F3: emotional representation), while AVE ranged from 0.23 (F1) to 0.50 for the best case (F8: timeline cyclical; AVE = 0.497). Most constructs met the CR ≥ 0.70 criterion (F2–F5 and F7–F9), whereas all AVE values were below 0.50 (with F8 borderline). Accordingly, convergent validity at the indicator level is limited; however, the pattern of statistically significant factor loadings and the theoretical coherence of the subscales support proceeding with the SEM analyses (see Table 4).

**TABLE 4:** Standardized factor loadings (*λ*) from confirmatory factor analysis (CFA).

Scale or items	*λ*
Self‐compassion	
Overidentification	0.395
Mindfulness	0.462
Isolation	0.544
Common humanity	0.492
Self‐kindness	0.476
Self‐judgment	0.480
Personal control	
Personal control_Q1	0.621
Personal control_Q2	0.645
Personal control_Q3	0.611
Personal control_Q4	0.631
Personal control_Q5	0.687
Personal control_Q6	0.686
Emotional representation	
Emotional representation_Q1	0.803
Emotional representation_Q2	0.823
Emotional representation_Q3	0.642
Emotional representation_Q4	0.567
Emotional representation_Q5	0.560
Emotional representation_Q6	0.668
Illness coherence	
Illness coherence_Q1	0.772
Illness coherence_Q2	0.634
Illness coherence_Q3	0.551
Illness coherence_Q4	0.560
Illness coherence_Q5	0.724
Treatment control	
Treatment control_Q1	0.610
Treatment control_Q2	0.599
Treatment control_Q3	0.764
Treatment control_Q4	0.763
Treatment control_Q5	0.591
Self‐care	
Self‐care maintenance	0.682
Self‐care monitoring	0.551
Self‐care management	0.607
Consequences	
Consequences_Q1	0.672
Consequences_Q2	0.759
Consequences_Q3	0.507
Consequences_Q4	0.694
Consequences_Q5	0.605
Consequences_Q6	0.568
Timeline cyclical	
Timeline cyclical_Q1	0.658
Timeline cyclical_Q2	0.711
Timeline cyclical_Q3	0.768
Timeline cyclical_Q4	0.677
Timeline acute	
Timeline acute_Q1	0.587
Timeline acute_Q2	0.746
Timeline acute_Q3	0.731
Timeline acute_Q4	0.638
Timeline acute_Q5	0.583
Timeline acute_Q6	0.689

*Note*: Values are standardized factor loadings (*λ*); all loadings were statistically significant (*p* < 0.001). *N* = 360.

Discriminant validity was examined using the Fornell–Larcker criterion. For all constructs, the square roots of AVE (√AVE ranged from 0.477 for self‐compassion to 0.705 for timeline cyclical) exceeded their absolute inter‐construct correlations (maximum observed *r* = 0.461 between self‐care and emotional representation), indicating adequate discriminant validity.

### 3.4 Model Fit and Direct‐Indirect Effects in Initial and Final SEM

The initial SEM, which included all hypothesized mediators, demonstrated an overall acceptable model fit (*χ* ^2^(1019) = 1623.40, *p* < 0.001; *χ* ^2^/df = 1.59; CFI = 0.882; TLI = 0.875; RMSEA = 0.041, 90% CI [0.037–0.044]; and SRMR = 0.082).

Examination of the standardized path coefficients revealed that several mediators demonstrated significant associations with self‐care, including personal control (*β* = 0.69, *p* < 0.001, 95% CI [−0.051, 0.427]), illness coherence (*β* = 0.31, *p* = 0.004, 95% CI [0.000, 0.498]), and emotional representation (*β* = 0.36, *p* = 0.001, 95% CI [0.059, 0.547]).

However, other hypothesized mediating pathways were not statistically significant. Specifically, the indirect effects of treatment control (*β* = –0.01, *p* = 0.893, 95% CI [−0.126, 0.071]), timeline acute/chronic (*β* = –0.34, *p* = 0.001, 95% CI [−0.241, 0.013]), timeline cyclical (*β* = –0.24, *p* = 0.018, 95% CI [−0.218, 0.002]), and consequences (*β* = –0.07, *p* = 0.443, 95% CI [−0.137, 0.022]) on self‐care did not reach significance. Bootstrapping analyses (5000 resamples) confirmed the non‐significance of mediation effects involving treatment control, timeline variables, and consequences (*p* > 0.05). Consequently, these variables were excluded from the model to improve parsimony and interpretability.

The refined (final) model also exhibited acceptable fit indices (*χ* ^2^(292) = 735.68, *p* < 0.001; *χ* ^2^/df = 2.52; CFI = 0.843; TLI = 0.825; RMSEA = 0.065, 90% CI [0.059–0.071]; and SRMR = 0.084).

**FIGURE 1:**
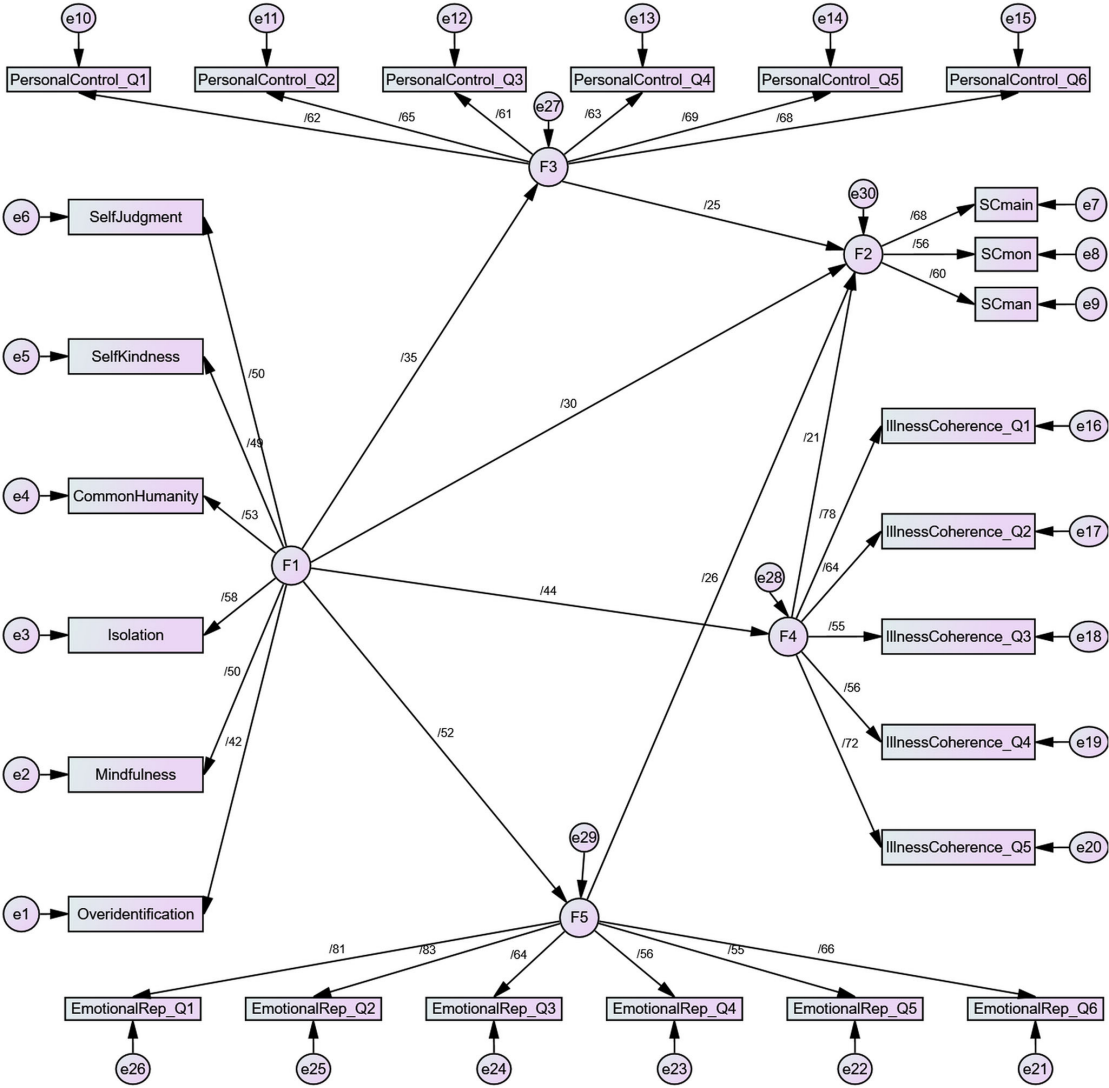
The model depicts self‐compassion as an independent latent variable influencing self‐care through multiple mediating illness perception factors such as personal control, illness coherence, and emotional representation. Standardized path coefficients illustrate the strength of these relationships.

In this final model, self‐compassion retained a significant direct effect on self‐care (*β* = 0.30, *p* = 0.006, 95% CI [0.107, 1.043]), along with significant indirect effects through personal control (*β* = 0.15, *p* = 0.004, 95% CI [0.043, 0.363]), emotional representation (*β* = 0.22, *p* = 0.011, 95% CI [0.060, 0.548]), and illness coherence (*β* = 0.15, *p* = 0.030, 95% CI [0.015, 0.450]). Together, these mediators accounted for a substantial portion of the total effect of self‐compassion on self‐care behaviors (see Figure 1).

## 4 Discussion

The present study investigated the relationship between self‐compassion and self‐care behaviors among individuals with MS, with particular emphasis on the mediating role of illness perceptions within Leventhal's CSM of illness representations. The findings demonstrated that self‐compassion was associated with self‐care both directly and indirectly through personal control, illness coherence, and emotional representation.

These results extend previous research by suggesting that the beneficial effects of self‐compassion in MS are not limited to emotional well‐being alone but also operate through illness‐related cognitive schemas that shape how patients interpret, regulate, and respond to the demands of chronic illness.

The observed direct association between self‐compassion and self‐care is consistent with a growing body of literature demonstrating that self‐compassion is positively associated with adaptive health behaviors across chronic illness populations. Previous studies have shown that individuals with higher self‐compassion tend to exhibit greater treatment adherence, healthier lifestyle behaviors, and improved emotional adjustment in conditions such as diabetes, cancer, and chronic pain (Zhu et al. 2020; Abdollahi et al. 2020; Ferrari et al. 2017; Widdrington et al. 2026).

Similar findings have also been reported in neurological conditions, including MS, where self‐compassion has been linked to lower psychological distress, reduced fatigue‐related suffering, and enhanced quality of life (Simpson et al. 2023; Eghbali et al. 2022). The present study expands this literature by demonstrating that self‐compassion is also significantly associated with behavioral self‐management in MS, reinforcing the idea that self‐compassion functions not merely as an emotional resilience factor but also as a facilitator of sustained health‐related action.

Importantly, the present study contributes to the literature by identifying specific dimensions of illness perception through which self‐compassion may influence self‐care behaviors. Whereas previous studies have often conceptualized illness perception as a broad and undifferentiated construct, the current findings suggest that only particular cognitive and emotional representations are functionally relevant in translating self‐compassion into self‐care behaviors in MS. More specifically, representations associated with cognitive integration, behavioral agency, and emotional regulation appear to play a central role in this relationship.

The significant direct association between self‐compassion and self‐care behaviors is particularly meaningful in the context of MS, a chronic neurological disorder characterized by unpredictability, progressive disability, fatigue, and recurrent psychological distress. Unlike acute illnesses in which health behaviors may depend primarily on short‐term symptom management, self‐care in MS requires sustained behavioral engagement despite fluctuating symptoms, uncertainty regarding disease progression, and considerable emotional burden (Luca et al. 2022). In such contexts, self‐compassion may function as an important psychological regulatory resource that enables patients to maintain adaptive behaviors even in the presence of setbacks, physical limitations, or treatment fatigue (Ueno and Amemiya 2024).

From a theoretical perspective, self‐compassion may facilitate self‐care by reducing maladaptive self‐evaluative processes such as shame, self‐criticism, and catastrophizing, which are common among individuals coping with chronic illness (Etemadi Shamsababdi and Dehshiri 2026). This interpretation aligns with Neff's conceptualization of self‐compassion as comprising self‐kindness, common humanity, and mindful awareness, all of which may buffer against emotional exhaustion and behavioral disengagement (Phillips and Hine 2021).

Patients with MS frequently experience disruptions in identity and perceived autonomy as a consequence of progressive functional impairment. Under these conditions, self‐critical responses may intensify distress and undermine motivation for self‐management (Özen et al. 2021). In contrast, self‐compassion promotes a more balanced and accepting stance toward illness‐related difficulties, thereby allowing patients to remain engaged in health‐promoting behaviors without becoming overwhelmed by failure or symptom fluctuations (Dahmardeh et al. 2021). Consequently, the relationship between self‐compassion and self‐care in MS appears not merely motivational but fundamentally regulatory in nature.

The mediating roles of personal control and illness coherence provide important empirical support for Leventhal's CSM, which conceptualizes illness behavior as the outcome of cognitive and emotional representations of disease. According to this framework, individuals actively construct beliefs regarding their illness—including its causes, controllability, timeline, and consequences—and these representations subsequently shape coping responses and health‐related behaviors (Hagger and Orbell 2022). Previous studies in MS and other chronic conditions have consistently shown that stronger perceptions of personal control and better illness understanding are associated with improved treatment adherence, adaptive coping, and better psychological adjustment. The present findings extend this literature by suggesting that self‐compassion may act as an upstream psychological resource that promotes these adaptive illness representations.

Individuals with greater self‐compassion may be less likely to respond defensively to illness‐related information and therefore more capable of developing coherent and integrated understandings of MS. Such cognitive clarity may enhance adherence to self‐care because patients who better understand symptom patterns, treatment rationales, and disease management strategies are more likely to perceive self‐care behaviors as meaningful and effective. This finding is consistent with prior research demonstrating that illness coherence predicts proactive disease management and reduced uncertainty‐related distress among individuals with chronic neurological conditions (Bassi et al. 2020).

Similarly, the mediating role of personal control indicates that self‐compassion may strengthen patients’ perceived agency in managing illness‐related challenges. Importantly, this does not necessarily imply unrealistic beliefs regarding control over disease progression. Rather, self‐compassion may foster adaptive forms of control centered on behavioral regulation, symptom management, and emotional coping. In chronic conditions such as MS, where complete control over disease outcomes is often unattainable, perceived control over daily self‐management behaviors may be psychologically more important than perceived control over the illness itself. This interpretation aligns with previous findings suggesting that perceived self‐efficacy and agency are among the strongest predictors of adherence and health behavior maintenance in MS populations (Davenport et al. 2024; Wilski et al. 2019).

The mediating role of emotional representation further underscores the importance of emotional processing mechanisms in chronic illness adaptation. Within the IPQ‐R framework, emotional representation reflects the extent to which patients experience negative emotional reactions such as fear, sadness, anger, or anxiety in response to illness (Hagger and Orbell 2022). Previous studies have shown that heightened illness‐related emotional distress is associated with poorer adherence, increased fatigue, and diminished quality of life in MS (Luca et al. 2022; Nery‐Hurwit et al. 2018). The present findings suggest that self‐compassion may reduce the intensity and behavioral impact of these emotional reactions.

This mechanism is particularly relevant in MS because emotional distress is often intertwined with symptom exacerbation and reduced treatment adherence. Patients with high illness‐related emotional reactivity may avoid disease management behaviors due to hopelessness, emotional exhaustion, or cognitive overload (Bassi et al. 2016). Self‐compassion, however, may facilitate emotional regulation through mindfulness and nonjudgmental awareness, enabling patients to acknowledge distress without becoming consumed by it (Sirois and Hirsch 2019). Importantly, these findings should not be interpreted as suggesting that self‐compassion eliminates negative emotions. Rather, self‐compassion may modify patients’ relationship with those emotions by reducing experiential avoidance and increasing tolerance for illness‐related distress (Eghbali et al. 2022). This distinction is theoretically significant because successful self‐care in chronic illness often depends not on the absence of distress, but on the ability to maintain goal‐directed behaviors despite emotional discomfort.

Although treatment control, timeline perceptions, and perceived consequences were associated with self‐compassion, these dimensions did not significantly mediate the relationship between self‐compassion and self‐care behaviors in the final structural model. Several explanations may account for these findings. First, treatment control may primarily reflect confidence in medical interventions rather than confidence in one's own behavioral capacity. This interpretation is consistent with prior research suggesting that beliefs about treatment effectiveness do not necessarily translate into active patient engagement in daily self‐management (Bassi et al. 2016). In the context of MS, where disease management relies heavily on pharmacological treatment and specialist care, beliefs regarding treatment effectiveness may operate relatively independently from everyday self‐care engagement.

Second, the nonsignificant role of timeline perceptions may reflect the unique phenomenology of MS as a chronic yet unpredictable illness characterized by relapses, remissions, and uncertain progression. Similar inconsistencies regarding the predictive role of timeline beliefs have been reported in previous CSM‐based studies, suggesting that perceiving an illness as chronic may provide limited actionable information relevant to behavioral self‐management. Such perceptions may function more as descriptive illness beliefs than as motivational determinants of self‐care behavior (Luca et al. 2022; Wilski et al. 2019).

Likewise, the perception of illness consequences did not significantly mediate the relationship between self‐compassion and self‐care, suggesting that awareness of illness severity alone may be insufficient to promote adaptive self‐management in the absence of perceived agency or emotional regulation. This finding aligns with prior studies indicating that threat appraisal can sometimes motivate behavior but may also provoke helplessness or avoidance when not accompanied by coping resources (Bassi et al. 2020, Bassi et al. 2016).

### 4.1 Limitations and Future Directions

Several limitations of the present study should be considered. First, the cross‐sectional design precludes causal inference, and therefore the observed relationships should be interpreted as correlational rather than causal. Longitudinal and interventional studies are needed to establish temporal directionality and determine whether increases in self‐compassion lead to subsequent improvements in illness perceptions and self‐care behaviors.

Second, the study did not control for important clinical variables such as MS subtype, disease activity status, or treatment regimen, all of which may influence illness perceptions and self‐management behaviors. Third, the absence of screening for depression and anxiety constitutes a notable limitation, particularly given the high prevalence of affective symptoms among individuals with MS and the inclusion of emotional representation as a key variable in the model.

Fourth, reliance on self‐report measures may have introduced response biases or social desirability effects. Future studies could benefit from multi‐method assessment strategies, including clinician evaluations, behavioral indicators, or longitudinal monitoring of self‐care practices. Additionally, the use of convenience sampling limits the generalizability of the findings, particularly within SEM contexts where sample representativeness is important. Future research should therefore employ random or stratified sampling procedures to improve external validity.

Finally, broader psychosocial variables such as coping styles, social support, resilience, and mental health status were not examined and may further clarify the mechanisms linking self‐compassion and self‐care in chronic illness populations. Future studies may also investigate whether compassion‐based interventions can directly modify illness perceptions and whether such changes mediate long‐term improvements in disease management.

### 4.2 Clinical Implications and Recommendations

Despite these limitations, the present findings have important clinical implications for improving self‐care among individuals with MS. The results suggest that fostering self‐compassion may enhance self‐care not only by reducing psychological distress but also by strengthening specific adaptive illness perceptions—particularly personal control, illness coherence, and emotional representation. In the context of MS, where sustained self‐management is often challenged by symptom unpredictability and emotional burden, self‐compassion may function as a self‐regulatory resource that helps patients remain engaged in health‐promoting behaviors despite setbacks or treatment fatigue.

Consistent with Leventhal's CSM, interventions may be most effective when they combine compassion‐based approaches with psychoeducation and self‐management support aimed at increasing patients’ sense of behavioral agency, improving their understanding of the illness, and reducing maladaptive emotional reactions to disease‐related stress.

Importantly, self‐compassion did not appear to influence all illness perception dimensions equally. Beliefs related to treatment control, disease chronicity, and perceived consequences were not significant mediators, suggesting that these dimensions may require alternative strategies such as targeted education, cognitive restructuring, or clinician‐guided communication. Taken together, these findings support a multidimensional intervention framework in which self‐compassion training is integrated with illness‐specific psychological and educational support to promote more effective long‐term disease management in MS.

## Author Contributions

**Zahra Ebadi**: data curation, project administration. **Abbas Masjedi‐arani**: supervision. **Ali Bagheri**: conceptualization, writing – original draft, formal analysis, methodology, writing – review and editing. **Maryam Amini‐Fasakhoudi**: project administration. **Amir Sam Kianimoghadam**: supervision.

## Funding

The authors have nothing to report.

## Conflicts of Interest

The authors declare no conflicts of interest.

## Supporting information




**Supplementary Material**: brb371772‐sup‐0001‐SuppMat.docx

**Supplementary Material**: brb371772‐sup‐0002‐SuppMat.docx

**Supplementary Material**: brb371772‐sup‐0003‐SuppMat.docx

**Supplementary Material**: brb371772‐sup‐0004‐SuppMat.docx

## Data Availability

The data supporting this study's findings are available from the corresponding author upon reasonable request.
