# Supplementary material for: The Role of Self‐Compassion on Self‐Care With the Mediating Role of Illness Perception in Patients With Multiple Sclerosis Referred to Sina Hospital: Based on the Leventhal Model
Source: Brain Behav. 2026 Sep 17;16(9):e71772. doi: 10.1002/brb3.71772 (PMC13586664; doi:10.1002/brb3.71772)
Supplement: Supplementary file 1 [file BRB3-16-e71772-s001.docx]

**Supplementary File 1: Linearity Assessment of Study Variables**

Scatterplot illustrating a positive linear relationship between self-compassion and personal control among study participants, with no evidence of nonlinear patterns.

Scatterplot depicting the relationship between self-compassion and perceived consequences, showing no clear linear or nonlinear pattern.

Scatterplot illustrating the association between self-compassion and emotional representation, with no evident nonlinear or curvilinear trends.

Scatterplot depicting the relationship between self-compassion and timeline acute, showing no evidence of nonlinear or curvilinear patterns.

Scatterplot illustrating the relationship between self-compassion and treatment control, indicating no observable nonlinear or curvilinear patterns.

Scatterplot showing the relationship between self-compassion and illness coherence, demonstrating no observable nonlinear or curvilinear patterns.

Scatterplot illustrating the relationship between self-compassion and timeline cyclical, showing no visible nonlinear or curvilinear trends.

Scatterplot depicting the relationship between self-care and timeline acute, illustrating a generally linear distribution without noticeable nonlinear patterns.

Scatterplot showing the relationship between self-care and consequences, indicating no clear nonlinear patterns and a generally random distribution of points.

Scatterplot illustrating the relationship between personal control and self-care, showing a generally random distribution of residuals and no indication of nonlinear patterns.

Scatterplot depicting the relationship between treatment control and self-care, indicating a random distribution of residuals with no evident nonlinear trends.

Scatterplot demonstrating the linear relationship between Illness Coherence and Self-Care, showing a relatively uniform distribution of data points without any nonlinear patterns.

Scatterplot illustrating the relationship between Timeline Cyclical and Self-Care, with data points evenly distributed and no indication of nonlinear trends.

Scatterplot depicting the relationship between Emotional Representation and Self-Care, showing a clear linear association with data points distributed along the range of values.
