# Supplementary material for: The Role of Self‐Compassion on Self‐Care With the Mediating Role of Illness Perception in Patients With Multiple Sclerosis Referred to Sina Hospital: Based on the Leventhal Model
Source: Brain Behav. 2026 Sep 17;16(9):e71772. doi: 10.1002/brb3.71772 (PMC13586664; doi:10.1002/brb3.71772)
Supplement: Supplementary file 2 [file BRB3-16-e71772-s002.docx]

**Supplementary File 2: Normality Assessment of Study Variables**

1. **Descriptive Statistics of Variables**

| Variable | Mean | Std. Deviation | Skewness | Kurtosis |
| --- | --- | --- | --- | --- |
| Self-Compassion | 3.009 | 0.353 | -0.006 | -0.127 |
| Timeline Acute | 3.101 | 0.537 | -0.304 | 0.256 |
| Consequences | 2.82 | 0.498 | 0.043 | 0.308 |
| Personal Control | 3.144 | 0.535 | -0.008 | 0.336 |
| Treatment Control | 3.146 | 0.545 | 0.031 | 0.049 |
| Illness Coherence | 3.156 | 0.492 | -0.096 | 0.253 |
| Timeline Cyclical | 2.914 | 0.552 | 0.283 | 0.467 |
| Emotional Representation | 3.123 | 0.493 | -0.028 | 1.084 |
| Self-Care | 3.265 | 0.422 | 0.037 | 0.236 |

## 2- Tests of Normality

| Variable | Kolmogorov-Smirnov Statistic | df | Sig. KS | Shapiro-Wilk Statistic | df_SW | Sig. SW |
| --- | --- | --- | --- | --- | --- | --- |
| Self-Compassion | 0.023 | 360 | 0.200 | 0.998 | 360 | 0.942 |
| Timeline Acute | 0.096 | 360 | 0.000 | 0.984 | 360 | 0.001 |
| Consequences | 0.088 | 360 | 0.000 | 0.987 | 360 | 0.003 |
| Personal Control | 0.086 | 360 | 0.000 | 0.986 | 360 | 0.002 |
| Treatment Control | 0.083 | 360 | 0.000 | 0.988 | 360 | 0.003 |
| Illness Coherence | 0.109 | 360 | 0.000 | 0.981 | 360 | 0.000 |
| Timeline Cyclical | 0.133 | 360 | 0.000 | 0.974 | 360 | 0.000 |
| Emotional Representation | 0.112 | 360 | 0.000 | 0.978 | 360 | 0.000 |
| Self-Care | 0.047 | 360 | 0.050 | 0.997 | 360 | 0.793 |

*Note: Kolmogorov-Smirnov significance for Self-Compassion is a lower bound estimate.

**Self-Compassion**

This histogram shows the frequency distribution of the self-compassion scores among participants, illustrating how data points are spread across the scale.

This plot compares the observed values against expected normal values to assess the normality of the distribution.

This plot shows deviations from the expected normal distribution to highlight any departures from normality.

**Timeline Acute**

Visualizes the frequency distribution of acute timeline perceptions among participants.

Compares observed values to a theoretical normal distribution to check for normality.

Highlights deviations from normality by plotting residuals from the expected distribution.

**Consequences**

Displays frequency of scores on the consequences subscale.

Assesses how closely the data follow a normal distribution.

Highlights deviations from normality.

**Personal Control**

Shows the distribution frequency of personal control scores.

Plots observed personal control scores against expected normal values.

Shows deviations from the expected normal distribution.

**Treatment Control**

Frequency distribution of treatment control perceptions.

Visual normality check comparing observed and expected values.

Displays deviations from the normal distribution.

**Illness Coherence**

Shows frequency distribution of illness coherence scores.

Normality assessment plot comparing observed vs. expected.

Highlights any departures from normality.

**Timeline Cyclical**

Distribution frequency of cyclical timeline perceptions.

Normality check plot.

Deviations from expected normal distribution.

**Emotional Representation**

Frequency distribution of emotional representation scores.

Plot assessing normality.

Deviations from normality shown.

**Self-Care**

Frequency distribution of self-care scores.

Normality assessment via expected vs. observed values.

Shows deviations from normality.
