# Supplementary material for: The Role of Self‐Compassion on Self‐Care With the Mediating Role of Illness Perception in Patients With Multiple Sclerosis Referred to Sina Hospital: Based on the Leventhal Model
Source: Brain Behav. 2026 Sep 17;16(9):e71772. doi: 10.1002/brb3.71772 (PMC13586664; doi:10.1002/brb3.71772)
Supplement: Supplementary file 3 [file BRB3-16-e71772-s003.docx]

**Supplementary File 4: Analysis of Residual Independence and Multicollinearity Analysis**

| Variables Entered | Variables Removed | Method |
| --- | --- | --- |
| Emotional Representation | . | Enter |
| Consequences |  |  |
| Timeline Cyclical |  |  |
| Personal Control |  |  |
| Treatment Control |  |  |
| Self-compassion |  |  |
| Timeline Acute |  |  |
| Illness Coherence^b^ |  |  |

a. Dependent Variable: selfcare	b. All requested variables entered.

**Variables Entered/Removed^a^.** This table lists the predictors included in the regression model for self-care, with all specified variables entered and none removed.

| Durbin-Watson | Std. Error of the Estimate | Adjusted R Square | R Square | R |
| --- | --- | --- | --- | --- |
| 1.472 | .33632 | .363 | .378 | .615^a^ |

a. Predictors: (Constant), Emotional Representation, Consequences, Timeline Cyclical, Personal Control, Treatment Control, self-compassion, Timeline Acute, Illness Coherence b. Dependent Variable: selfcare

**Model Summary^b^.** The model explains a substantial proportion of variance in self-care, indicated by an R of .615 and an R² of .378. The Durbin-Watson statistic suggests no serious autocorrelation in residuals.

| Sig. | F | Mean Square | df | Sum of Squares | Model |
| --- | --- | --- | --- | --- | --- |
| .000^b^ | 26.623 | 3.011 | 8 | 24.091 | Regression |
|  |  | .113 | 351 | 39.701 | Residual |
|  |  |  | 359 | 63.792 | Total |

a. Dependent Variable: selfcare b. Predictors: (Constant), Emotional Representation, Consequences, Timeline Cyclical, Personal Control, Treatment Control, self-compassion, Timeline Acute, Illness Coherence

**ANOVA^a^.** The ANOVA table confirms the overall significance of the regression model in predicting self-care (p < .001).

| Model | Unstandardized Coefficients B | Std. Error | Standardized Coefficients Beta | t | Sig. | Collinearity Statistics | | |
| --- | --- | --- | --- | --- | --- | --- | --- | --- |
|  |  |  |  |  |  | Tolerance | VIF | |
| (Constant) | 1.433 | .217 |  | 6.609 | .000 |  |  | |
| Emotional Representation | .195 | .054 | .164 | 3.588 | .000 | .850 | 1.176 | |
| Consequences | -.131 | .038 | -.167 | -3.487 | .001 | .772 | 1.295 | |
| Timeline Cyclical | -.008 | .038 | -.010 | -.213 | .831 | .864 | | 1.157 |
| Personal Control | .166 | .037 | .211 | 4.511 | .000 | .809 | | 1.236 |
| Treatment Control | .013 | .036 | .016 | .350 | .727 | .808 | | 1.237 |
| Self-compassion | .180 | .042 | .210 | 4.306 | .000 | .742 | | 1.347 |
| Timeline Acute | -.067 | .035 | -.087 | -1.881 | .061 | .824 | | 1.213 |
| Illness Coherence^b^ | .235 | .042 | .275 | 5.560 | .000 | .723 | | 1.383 |

a. Dependent Variable: selfcare

**Coefficients^a^.** Regression coefficients indicate the direction, magnitude, and significance of each predictor's effect on self-care. Variables such as Emotional Representation, Personal Control, Self-Compassion, and Illness Coherence have significant positive effects, while Consequences has a significant negative effect. Tolerance and VIF values indicate no problematic multicollinearity among predictors.

|  | Minimum | Maximum | Mean | Std. Deviation | N |
| --- | --- | --- | --- | --- | --- |
| Predicted Value | 2.3607 | 4.1223 | 3.2645 | .25905 | 360 |
| Residual | -1.21457 | 1.04273 | .00000 | .33255 | 360 |
| Std. Predicted Value | -3.489 | 3.311 | .000 | 1.000 | 360 |
| Std. Residual | -3.611 | 3.100 | .000 | .989 | 360 |

a. Dependent Variable: selfcare

**Residuals Statistics^a^.** Descriptive statistics for predicted values and residuals show an approximately normal distribution supporting model assumptions.

| Dimension | Eigenvalue | Condition Index | (Constant) | Self-compassion | Timeline Acute | Consequences | Personal Control | Treatment Control | Illness Coherence | Timeline Cyclical | Emotional Repression |
| --- | --- | --- | --- | --- | --- | --- | --- | --- | --- | --- | --- |
| 1 | 8.843 | 1.000 | .00 | .00 | .00 | .00 | .00 | .00 | .00 | .00 | .00 |
| 2 | .039 | 14.975 | .00 | .02 | .12 | .05 | .08 | .04 | .05 | .14 | .07 |
| 3 | .025 | 18.810 | .00 | .00 | .01 | .52 | .01 | .00 | .00 | .52 | .00 |
| 4 | .023 | 19.508 | .00 | .01 | .07 | .22 | .15 | .36 | .00 | .19 | .03 |
| 5 | .019 | 21.520 | .01 | .06 | .05 | .00 | .37 | .41 | .00 | .04 | .14 |
| 6 | .018 | 22.362 | .00 | .03 | .67 | .10 | .24 | .15 | .00 | .09 | .00 |
| 7 | .015 | 24.267 | .02 | .18 | .01 | .01 | .12 | .00 | .73 | .00 | .00 |
| 8 | .013 | 26.572 | .05 | .17 | .01 | .07 | .02 | .02 | .19 | .00 | .74 |
| 9 | .005 | 41.090 | .92 | .54 | .06 | .02 | .01 | .01 | .02 | .02 | .01 |

**Collinearity Diagnostics.** Condition indices and variance proportions reveal no severe multicollinearity issues, as all values fall within acceptable ranges.
