# Supplementary material for: The Role of Self‐Compassion on Self‐Care With the Mediating Role of Illness Perception in Patients With Multiple Sclerosis Referred to Sina Hospital: Based on the Leventhal Model
Source: Brain Behav. 2026 Sep 17;16(9):e71772. doi: 10.1002/brb3.71772 (PMC13586664; doi:10.1002/brb3.71772)
Supplement: Supplementary file 4 [file BRB3-16-e71772-s004.docx]

**Supplementary File 3: Assessment of Homoscedasticity (Variance Homogeneity)**

**TimelineAcute**

Scatterplot depicting the standardized residuals versus standardized predicted values for the regression model predicting self-care. The residuals appear randomly and evenly distributed around zero, supporting the assumptions of homoscedasticity and linearity in the model.

**Consequences**

This scatterplot illustrates the standardized residuals against the standardized predicted values of the regression model. The points are fairly evenly dispersed around the zero line, indicating a balanced distribution of residuals and the absence of any discernible pattern, thereby supporting the assumptions underlying the regression model.

**Personal Control**

Scatterplot showing the standardized residuals versus standardized predicted values for the regression model. The residuals display a random pattern with no evident heteroscedasticity, supporting model assumptions of variance homogeneity.

**Treatment Control**

Standardized residuals plotted against standardized predicted values for the treatment control variable within the mediation model. Residuals are evenly spread, indicating homoscedasticity and appropriate model fit.

**IllnessCoherence**

Scatterplot of standardized residuals versus predicted values, illustrating no pattern or systematic variance change, thereby supporting homoscedasticity in the model.

**Timeline Cyclical**

Plot showing residuals distribution for the cyclical timeline mediator variable. Residuals are randomly scattered with no indication of variance heterogeneity.

**Emotional Representation**

Standardized residuals plotted against predicted values show no funnel shape or clustering, confirming the homogeneity of variance assumption.

**Self-compassion**

Scatterplot of residuals versus predicted values for the independent variable self-compassion. The residuals are randomly distributed around zero, supporting the homoscedasticity assumption necessary for regression analyses.
